# Supplementary material for: Causes and consequences of sex-chromosome turnovers in Diptera
Source: Evol Lett. 2026 Mar 12;10(3):253–68. doi: 10.1093/evlett/qrag003 (PMC13231274; doi:10.1093/evlett/qrag003)
Supplement: qrag003_Supplemental_Files [file qrag003_supplemental_files.zip › Supplementary_Datasets.zip!/Supplementary_Datasets/Dataset_2_Graphical_representation_outgroups/Lauxaniidae_Calliopum_simillimum_chromosomes.pdf]

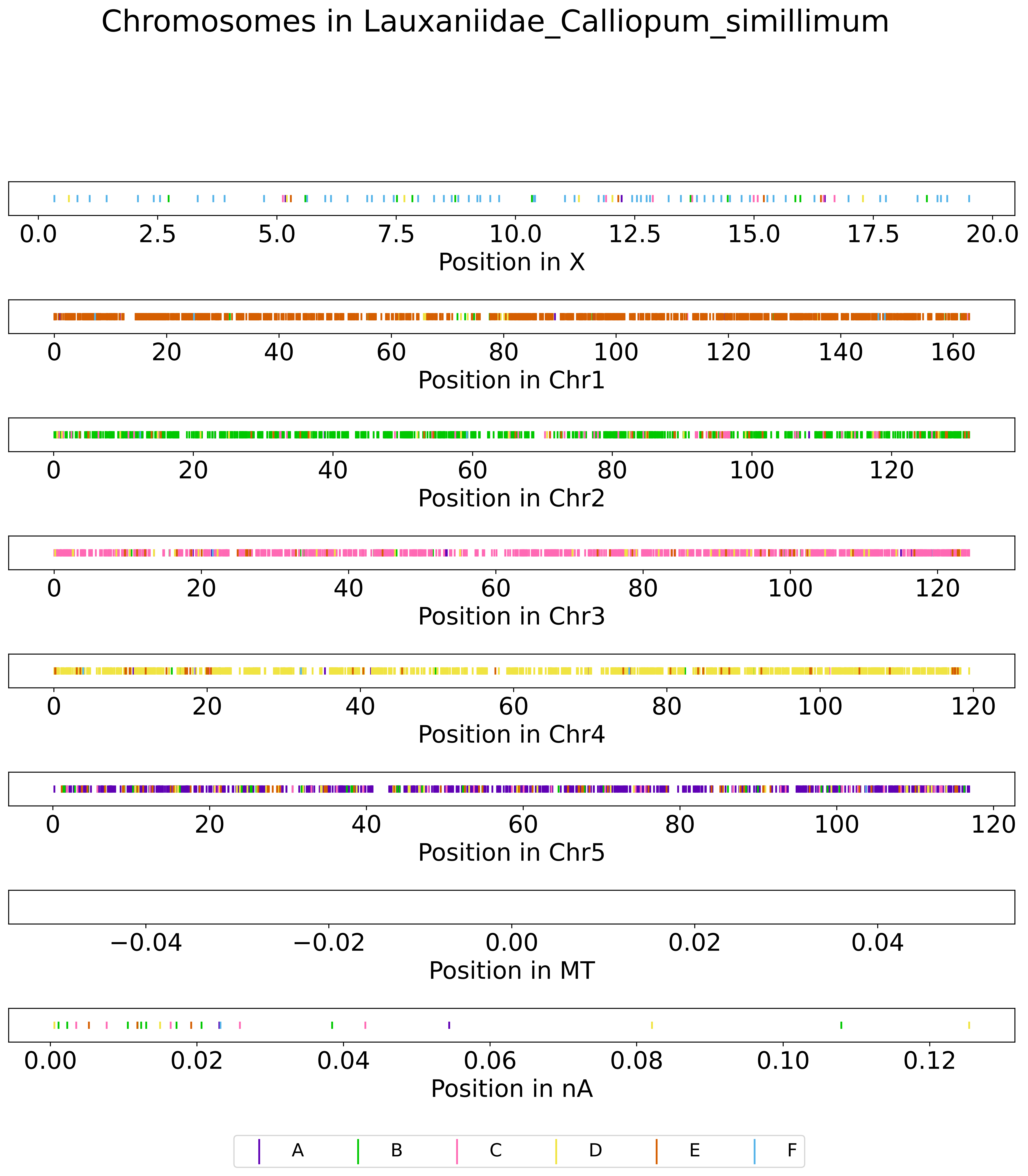
Chromosomes in Lauxaniidae_Calliopum_simillimum
0.0 2.5 5.0 7.5 10.0 12.5 15.0 17.5 20.0
Position in X
0 20 40 60 80 100 120 140 160
Position in Chr1
0 20 40 60 80 100 120
Position in Chr2
0 20 40 60 80 100 120
Position in Chr3
0 20 40 60 80 100 120
Position in Chr4
0 20 40 60 80 100 120
Position in Chr5
−0.04 −0.02 0.00 0.02 0.04
Position in MT
0.00 0.02 0.04 0.06 0.08 0.10 0.12
Position in nA
A
B
C
D
E
F
